# Numerical and Experimental Approaches for Mechanical Durability Assessment of an EV Battery Pack Case

**DOI:** 10.3390/ma18245683

**Published:** 2025-12-18

**Authors:** Hyun Soo Kim, Mingoo Cho, Changyeon Lee, Jaewoong Kim, Sungwook Kang

**Affiliations:** 1 Jinju Center, Dongnam Technology Application Division, Korea Institute of Industrial Technology, Jinju-si 52845, Republic of Korea; hyun0702@pusan.ac.kr (H.S.K.); cmg0142@kitech.re.kr (M.C.); 2 School of Mechanical Engineering, Pusan National University, Busan 46241, Republic of Korea; 3 R&D Center, Daejoo Kores Company, Wanju 55316, Republic of Korea; cyeonlee@daejookc.com; 4 Purpose Built Mobility Group, Korea Institute of Industrial Technology, Gwangju 61012, Republic of Korea; 5 Department of Smart Ocean Mobility Engineering, Changwon National University, Changwon-si 51140, Republic of Korea

**Keywords:** battery pack case, electric vehicles, mechanical durability, structural integrity, finite element analysis, experimental validation

## Abstract

Electric vehicle (EV) battery pack cases (BPCs) must withstand mechanical loads such as impact, compression, and vibration to ensure structural integrity and passenger safety. This study evaluates the mechanical durability of a full-scale aluminum BPC using combined experimental testing and finite element analysis (FEA). A bottom impact test, 200 kN compression test, and power spectral density (PSD)-based random vibration test were conducted to simulate representative operating and handling conditions. The numerical model replicated boundary conditions and load profiles identical to the experiments, enabling a direct comparison of stress distribution and deformation characteristics. The results demonstrated that stress and displacement trends predicted by FEA closely matched experimental observations, with stress concentrations appearing at corner and frame junction regions and less than 1 mm deformation recorded under peak compression loading. Vibration responses were most pronounced in the vertical direction, without bolt loosening or structural damage. These results verify the reliability of the proposed BPC design and provide quantitative evidence supporting simulation-driven lightweight battery enclosure development.

## 1. Introduction

The widespread adoption of electric vehicles (EVs) is accelerating the transition toward sustainable transportation in response to demands such as those for addressing climate change, achieving carbon neutrality, and reducing reliance on fossil fuels. EVs produce no local exhaust emissions and can substantially lower their total lifecycle greenhouse gas (GHG) footprint when charged with renewable energy, making them a more environmentally friendly alternative to traditional internal combustion engine vehicles (ICEVs) [1]. Additionally, EVs have simpler structures, are more energy-efficient than ICEVs, and offer superior user convenience, such as quiet operation and rapid responsiveness. Due to these advantages, EVs are regarded as a core mobility technology capable of replacing ICEVs. At the heart of EVs lies the battery system, which requires high levels of safety and reliability. The battery system in an EV stores energy and supplies power to the vehicle, and its performance and durability directly affect the vehicle’s overall lifespan and safety. The battery pack, comprising cells and modules, is especially vulnerable as it is exposed to various external environments and loads. The surrounding battery pack case (BPC) does not merely protect the battery; it also serves multiple functions. As an integral part of the vehicle’s underbody structure, it must withstand significant mechanical, thermal, and vibrational loads [2,3]. In addition, the BPC plays a role in absorbing external impacts, mitigating vibrations, insulating against heat, and preventing fire propagation. Therefore, meeting these diverse functional demands simultaneously necessitates research based on quantitative and systematic design. Research on BPC has primarily progressed in four directions. First, material-based studies were conducted that focused on achieving both mechanical strength and lightweight design using high-strength aluminum alloys, high-tensile steel sheets, and composite materials (e.g., SMC, CFRP) [4,5,6,7,8]. Second, fundamental studies using the finite element method (FEM) were implemented, including structural response analysis as well as investigations into thermal flow characteristics, cooling performance optimization, and evaluation of material properties [9,10,11,12,13,14,15,16,17,18,19,20]. Third, experimental-validation studies where analysis results are confirmed through real-world crash and compression tests aimed at optimizing design based on validated FEM outcomes [21,22,23]. Fourth, advancement and enhanced accuracy in analysis techniques were introduced that transcend traditional static analysis to include methods such as explicit dynamics for high-speed impact response, frequency response evaluation based on modal analysis, and power spectral density (PSD)-based random response analysis. These advanced techniques enable more precise predictions of BPC behavior under real-world vehicle conditions and allow for more systematic performance validation through early design-stage simulations [24,25,26]. Recently, the significance of compression and vibration durability analysis has become more widely recognized. Compression analysis has become essential for evaluating the structural response of battery packs to compressive loads, such as those experienced during collisions. Analyses now incorporate not only uniaxial static loading conditions but also multiaxial loads and time-history-based pressure application scenarios. These analyses enable detailed assessments of structural responses, including inelastic behavior, local buckling, yielding, and fracture. One widely adopted approach is to use FEM simulations of loading through a rigid plate at a constant velocity to assess structural vulnerability by analyzing stress history, deformation, and residual stress [27,28,29]. In the case of vibration analysis, ensuring vibration durability has become a crucial research focus because high-frequency and repetitive loads experienced during various driving conditions can lead to long-term fatigue damage in battery systems. Dynamic loads arising from road irregularities, surface friction, and powertrain vibrations create structural responses across a wide frequency range, and PSD-based analysis techniques are commonly utilized to evaluate these effects. Input data for these analyses are typically configured as triaxial vibration histories or frequency response spectra, allowing for the quantitative evaluation of phenomena such as resonance, resonance amplification, and fatigue accumulation [30,31,32]. Given that EV battery systems are generally located underneath the vehicle, they are particularly vulnerable to road irregularities, obstacles, and underbody impacts in the event of an accident. Such impacts can result in mechanical damage that may lead to internal short circuits or thermal runaway, underscoring the need for structural reinforcement. As a result, both analytical and experimental studies have been actively conducted to assess resistance to underbody impacts and energy absorption capability, with various structural optimization strategies (such as multilayer protection structures and honeycomb panels) being proposed [33,34,35]. Research on thermal management and fire safety has also been actively conducted, with proposed techniques including the application of phase change materials (PCM), heat-resistant mica pads, and high-temperature-resistant composites to block heat conduction and suppress thermal runaway. Similar efforts have been made to model the thermal environment of BPCs using thermal flow analysis [36,37,38,39]. However, most prior studies have predominantly focused on thermal management-related analysis, such as cooling system design, cell arrangement, and thermal distribution optimization, while integrated analysis and testing aimed at ensuring the structural durability of BPCs remain relatively limited. Therefore, this study aims to address this gap by conducting a comprehensive structural reliability evaluation based on FEM simulations that integrate compression, bottom impact, and vibration analyses, complemented by experimental validation to ensure the reliability of the analysis results. The materials used include high-strength aluminum alloy (A6061), high-tensile steel sheets (SGACUD 60/60), and polyamide composite (PA6-GF30). The mechanical properties of each material were incorporated into the FEM model to enhance simulation accuracy [40,41,42]. The analysis conditions were defined based on scenario-driven parameters that reflect real-world driving and crash situations. Comprehensive analyses were performed, including structural vulnerability assessments, stress distribution, deformation analysis, and fatigue response under repeated loading. A full-scale BPC specimen designed for vehicles currently under development was subjected to standardized bottom impact, compression, and vibration tests. FEM analysis was conducted under identical conditions, enabling a quantitative comparison between the experimental results and the simulated outcomes, thereby validating the model’s reliability. In conclusion, this study validates the structural durability and safety of EV BPCs from multiple perspectives and presents design optimization strategies suitable for real-vehicle applications. The FEM-based findings provide foundational data for developing safety strategies that include thermal runaway mitigation, vibration durability assurance, and structural reinforcement design. By integrating FEM analysis with physical testing, it is anticipated that the results will contribute to enhancing the structural integrity of EV battery systems [43,44,45,46].

## 2. FEA Model and Structural Test Setup for the EV BPC

### 2.1. Analysis and Experimental Conditions for BPC Bottom Impact

**Figure 1:**
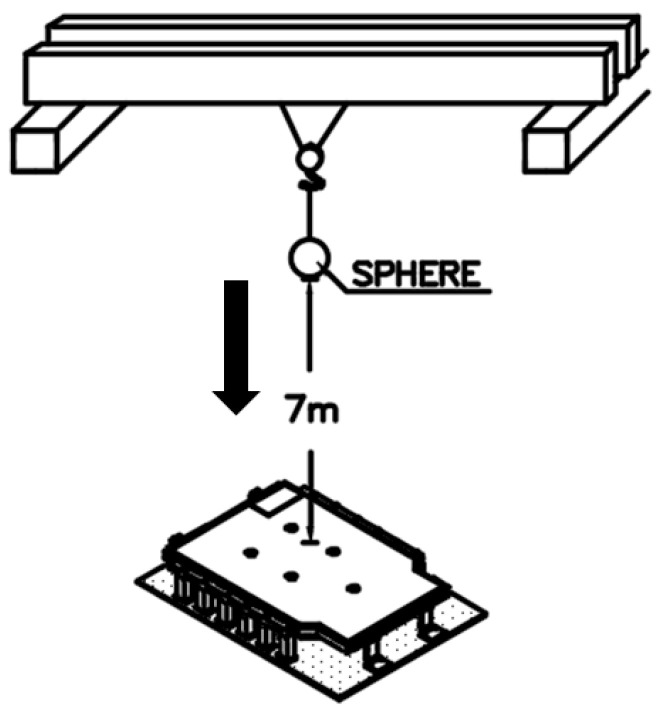
Experimental configuration for the bottom impact test conducted from a height of 7 m using a crane-based free fall setup.

In this study, a bottom impact test was conducted to evaluate the safety of an EV BPC. The full-scale BPC prototype used in this study was developed and fabricated by Daejoo Kores Co., Ltd. (Wanju, Jeollabuk-do, Republic of Korea), a company specializing in the design and manufacturing of EV battery enclosures. As shown in Figure 1, the test involved dropping an impactor from a height of 7 m. Prior to the physical test, FEA was performed under identical conditions to predict the load-transfer path and the influence of boundary conditions. The three-dimensional geometry of the BPC was modeled using ANSYS SpaceClaim 2024 R2 (ANSYS Inc., Canonsburg, PA, USA), and the finite-element analyses were performed in ANSYS Workbench 2024 R2. A hybrid mesh configuration combining hexahedral and tetrahedral solid elements was employed to accurately represent both flat and curved regions of the structure. Local mesh refinement was applied to the corner and frame-joint regions to enhance stress accuracy, and a convergence verification was conducted by varying mesh densities until the stress–strain responses stabilized within 2%. The results were then compared with those of the experiment. In the simulation, the impactor was modeled as a rigid body, and general contact conditions were applied. The BPC was fixed at the bottom, and both the elastic and plastic behaviors of its constituent materials were considered. Two types of impactors, both made of structural steel, were used for the bottom-impact tests: one spherical and one cylindrical. Each impactor weighed 10 kg. The tests were conducted following relevant domestic and international standards, including the Guidelines for Vehicle Underbody Impact Tests issued by the Ministry of Land, Infrastructure and Transport (MOLIT) [47], GB/T 31467.3 [48], and UN 38.3 [49]. Specifically, UN 38.3 [49] involves a 1.2 m drop test for individual cells and modules to ensure transportation safety, while GB/T 31467.3 [48] provides mechanical-shock-resistance criteria for battery systems. In Korea, MOLIT guidelines recommend drop heights of 2 m–5 m for impact testing. However, this study adopted a more stringent 7 m drop height to quantitatively assess the extreme durability of the BPC. FEA was conducted using an explicit-dynamics nonlinear approach. The results yielded the maximum deformation magnitude and location, stress-concentration zones, reaction-force history over time, and energy-absorption characteristics. This analysis enabled a quantitative assessment of the BPC’s structural vulnerability to bottom-impact loads. Finally, the simulation results were compared with those from the physical impact tests performed under the same conditions to validate the reliability and accuracy of the numerical model.

**Figure 2:**
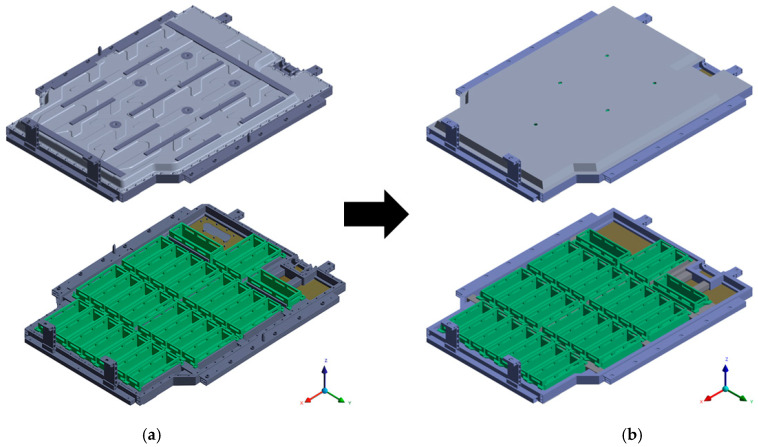
Comparison of battery pack case (BPC) models: (**a**) Detailed 3D CAD model including structural frames and internal layout. (**b**) Simplified model for finite element analysis with non-critical features removed.

**Figure 3:**
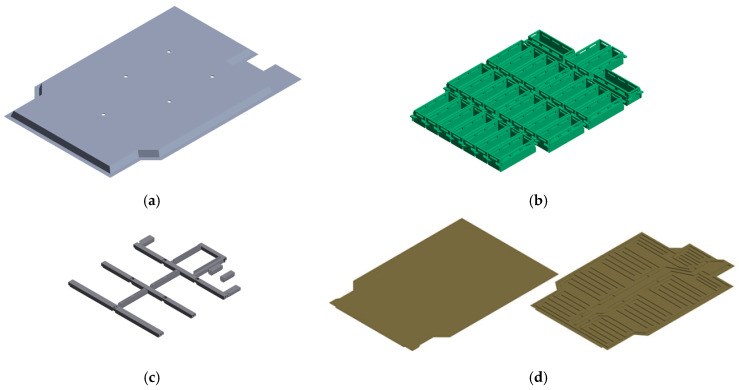

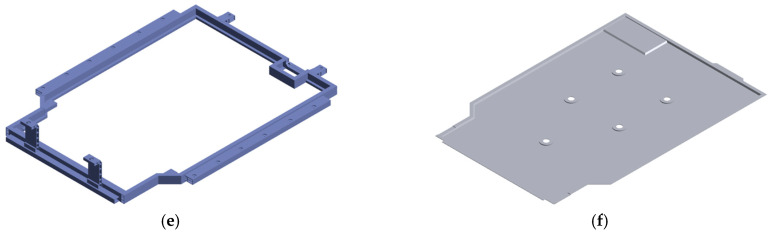
Component breakdown of the battery pack case (BPC) with material assignments: (**a**) SGACUD 60/60, (**b**) PA6-GF30, (**c**) A6082S-T6, (**d**) A3003-O, (**e**) A6N01S-T6, (**f**) A6061P.

**Figure 4:**
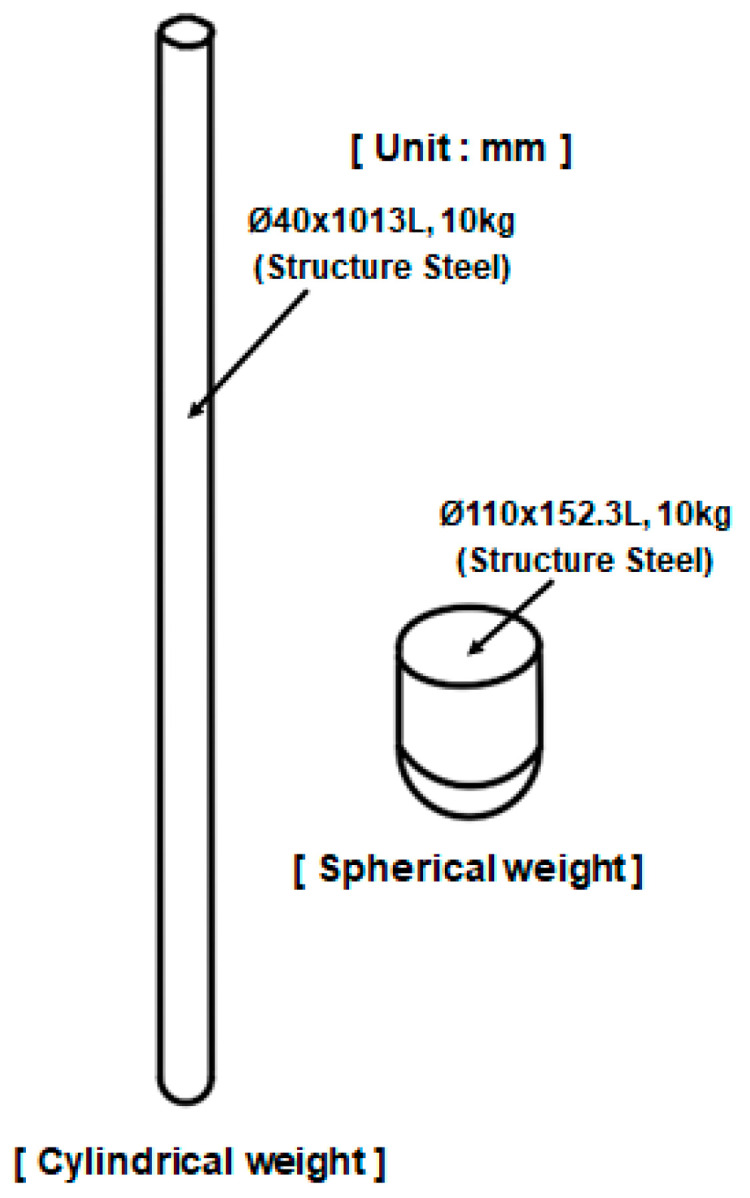
10 kg cylindrical and spherical impact weights used for the bottom impact test, made of structural steel.

Figure 2 compares the detailed 3D CAD model of the BPC with a simplified model used for FEA. Figure 2a shows the detailed CAD model, constructed based on actual manufacturing specifications. It accurately represents complex geometric features, such as fasteners, detailed shapes, and clearances between components. Figure 2b, on the other hand, presents the simplified model, where non-critical geometric features were removed to improve computational efficiency. Only the main load transfer paths and primary structural components were retained. This simplified model was designed to reduce computational cost while maintaining sufficient accuracy for predicting structural behavior. As a result, it allowed for an effective balance between simulation efficiency and analytical precision in the FEA process. Figure 3 shows the component breakdown of the BPC with material assignments in an exploded-view format. The components and their corresponding materials are: (a) high-tensile steel sheet SGACUD 60/60 (Hyundai Steel, Incheon, Republic of Korea), (b) PA6-GF30 composite (Lotte Chemical, Seoul, Republic of Korea), (c) aluminum alloy A6082S-T6 (Daejoo Kores, Wanju, Jeollabuk-do, Republic of Korea), (d) thermally conductive aluminum plate A3003-O (Daejoo Kores, Wanju, Jeollabuk-do, Republic of Korea) serving as both a cell-bottom protection plate and a cooling channel, (e) extruded aluminum A6N01S-T6 (Daejoo Kores, Wanju, Jeollabuk-do, Republic of Korea), and (f) bottom plate aluminum A6061P (Daejoo Kores, Wanju, Jeollabuk-do, Republic of Korea). The material properties used in FEA were assigned based on the numerical values listed in Table 1. Clearly defining the application locations of each material enabled a precise simulation of the BPC’s overall mechanical properties and local behavior characteristics. Figure 4 shows the shapes and material specifications of the impactors used in both the bottom-impact tests and the corresponding simulations. Two types of impactors were designed: a cylindrical impactor (Ø40 × 1013 mm) and a spherical impactor (Ø110 × 152.3 mm). Both impactors weighed 10 kg and were fabricated using structural steel (Hyundai Steel, Incheon, Republic of Korea). The same configurations were consistently applied in both the physical tests and numerical analyses. This dual-impactor approach enabled the comparison of contact characteristics and impact response sensitivity based on geometry, thereby facilitating an assessment of the BPC’s structural integrity and durability under various impact conditions.

**Figure 5:**
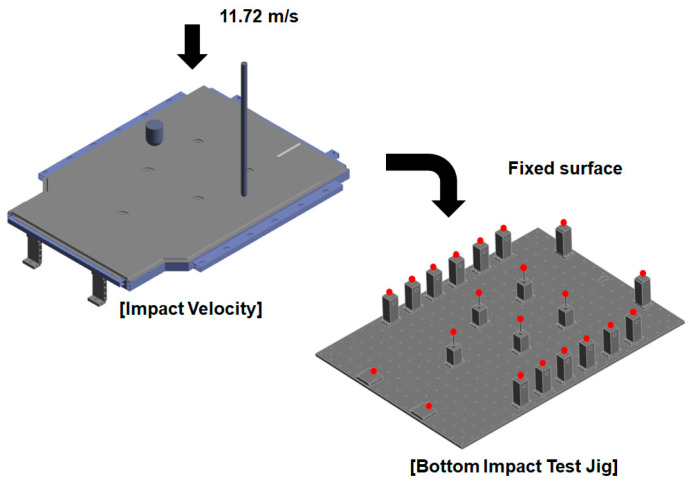
Finite element simulation setup for bottom impact analysis, simulating a 7 m free-fall of a rigid impactor striking the BPC at 11.72 m/s onto a fixed support jig.

The simulation conditions are illustrated in Figure 5, where the BPC was fixed to the lower jig, and a rigid impactor was dropped from above to strike it at a free-fall impact velocity of 11.72 m/s. The impact velocity *v* was calculated using gravitational acceleration *g* and the drop height *h* = 7 m, as expressed in Equation (1):

(1) $$v=\sqrt{2gh}$$

**Figure 6:**
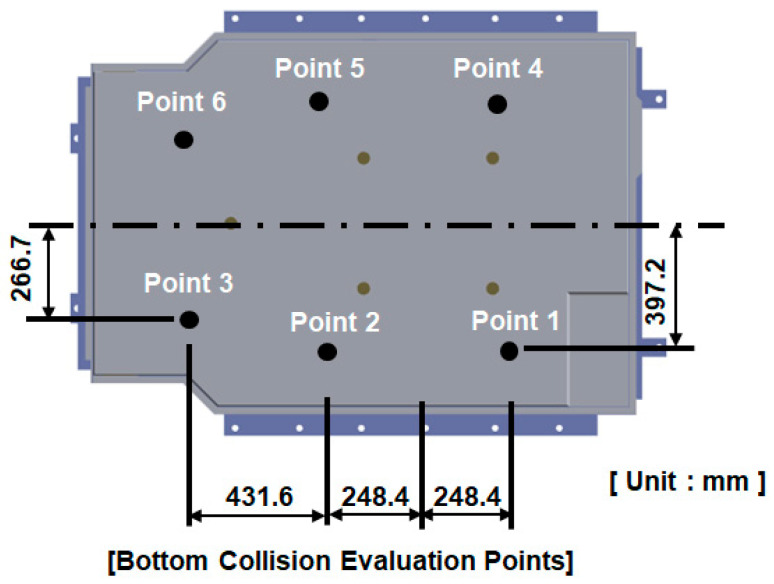
Designated impact points on the BPC top surface for bottom collision testing, corresponding to 7 m free-fall conditions, with dimensions in millimeters.

As shown in Figure 6, six bottom impact points were defined in the analysis. Cylindrical impactors were applied to Points 1–3, while spherical impactors were used at Points 4–6 to examine the differences in structural response depending on impactor geometry.

**Figure 7:**
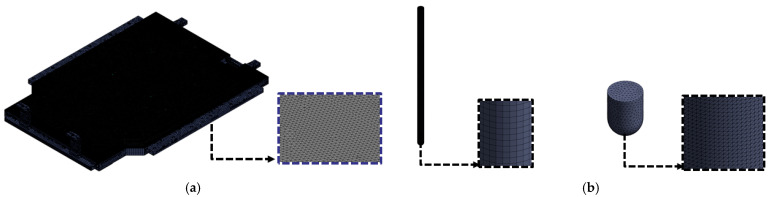
Finite element mesh models for bottom impact analysis: (**a**) battery pack case (BPC); (**b**) two rigid impactors (cylindrical and spherical) used as impact heads.

The BPC model and the two types of impactors (cylindrical and spherical) used in the analysis are depicted in Figure 7, with a summary of the finite element mesh information provided in Table 2. The complete BPC model consisted of approximately 852,867 nodes and 1,436,879 elements, while the cylindrical and spherical impactors comprised 27,600 and 15,865 nodes, respectively. To improve computational efficiency, both impactors were modeled as rigid bodies and were subject to general contact conditions. All constituent materials were represented with nonlinear material properties, including Young’s modulus, yield strength, and Poisson’s ratio, as listed in Table 1. Structural steel was used for the impactors and the fixed jig, whereas high-strength aluminum alloys and composite materials were applied to the BPC body to simulate actual assembly conditions. These settings enabled the bottom impact analysis to quantitatively evaluate the structural response of the BPC under various impact locations and impactor geometries. The results were subsequently compared with those from physical drop tests to validate the accuracy of the FEA model.

**Figure 8:**
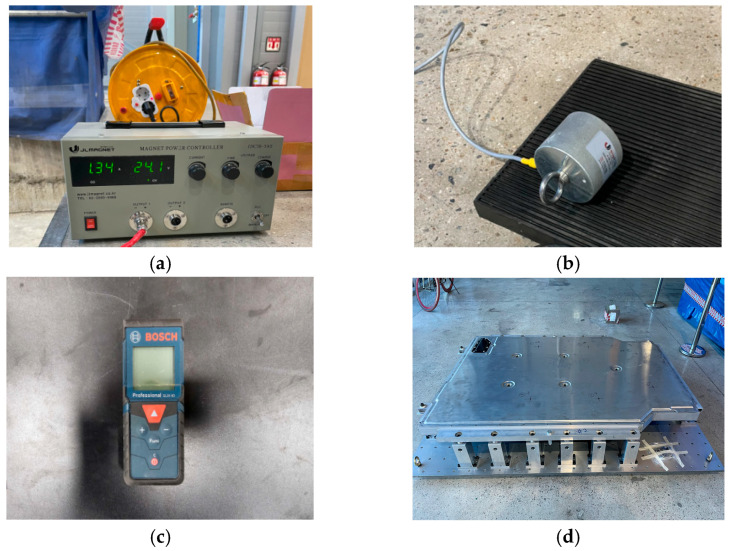

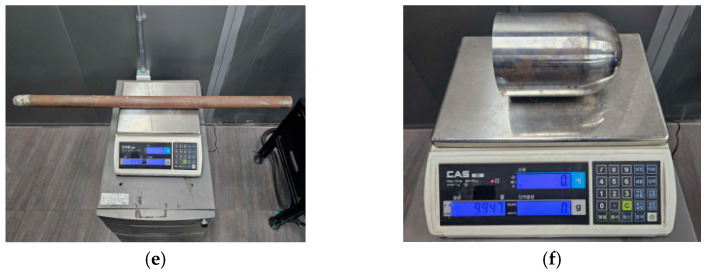
Experimental instrumentation and validation tools: (**a**) magnetic power controller, (**b**) magnetic holding unit, (**c**) laser distance meter, (**d**) BPC mounted on bottom impact jig, (**e**) cylindrical impactor on scale, (**f**) spherical impactor on scale.

Figure 8 shows the main equipment and instrumentation used for the bottom impact test. Figure 8a shows the magnetic power controller, which regulates the release of the impactor, with adjustable magnetic field strength based on the set current. Figure 8b shows the magnetic holding unit that secures the impactor in place until the test begins. The holding unit releases the impactor by deactivating the magnetic force at a predetermined moment, which initiates a free-fall impact. Figure 8c shows the laser distance meter, used for precise measurement of the drop height. The distance between the impact point and the surface of the fixed jig was measured in millimeters. Figure 8d shows the BPC mounted on the bottom impact test jig, reproducing the contact conditions between the BPC and the supporting structure for impact transmission. Figure 8e,f show the measured masses of the cylindrical and spherical impactors, respectively, obtained using an electronic balance. Both impactors were confirmed to have an exact mass of 10 kg. These procedures were conducted to ensure physical consistency between the numerical simulation and the experimental setup. The overall experimental configuration established a reliable and reproducible foundation for the bottom impact test, thus facilitating a quantitative comparison with the numerical analysis results.

### 2.2. FEA and Experimental Conditions for BPC Under Compression Load

**Figure 9:**
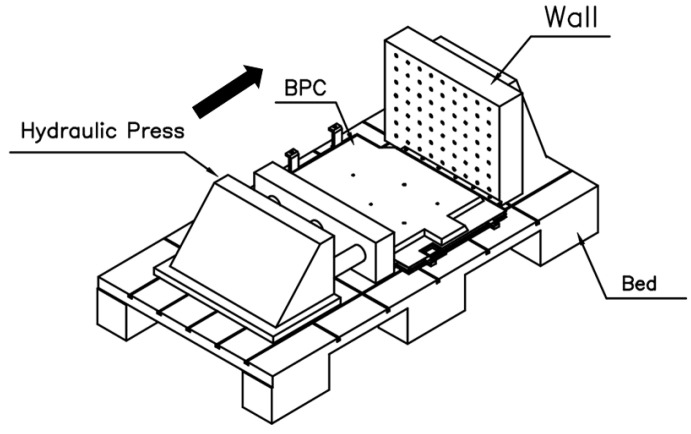
Schematic diagram of the compression test setup, showing the BPC specimen placed on the bed and constrained against a fixed wall while compression is applied via hydraulic press using flat crush plates.

**Figure 10:**
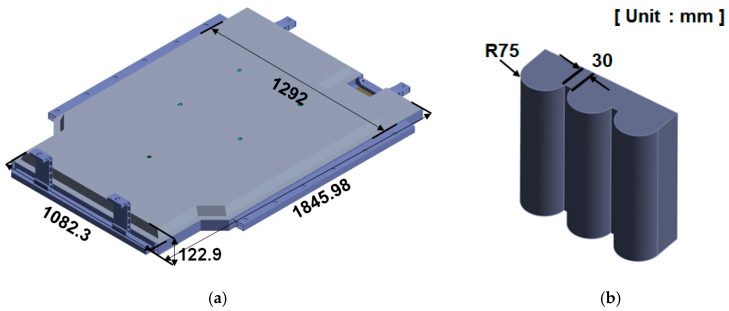
Geometric configuration for compression test setup: (**a**) battery pack case (BPC) with key dimensions in millimeters, (**b**) cylindrical crush plate used for compression testing (radius: 75 mm, width: 30 mm).

As shown in Figure 9, the experimental compression setup consisted of a hydraulic press applying load to the BPC while one side was constrained against a fixed wall. This configuration was replicated in both the numerical model and the physical test to ensure consistent boundary conditions. FEA was performed to assess the compressive stiffness and structural safety of the EV BPC. The BPC was made of aluminum alloy A6061-T6, and a 3D model was constructed to replicate the geometry of the test specimen. As shown in Figure 10a, the BPC’s external dimensions were 1845.98 mm (length) × 1292 mm (width) × 122.9 mm (height). It was positioned on a lower bed with one side constrained against a wall, and a compressive load was applied under these boundary conditions. Both the numerical analysis and the experimental tests were performed in accordance with the international standards UL 2596 [50] and GB/T 31467.3 [48], considering a maximum compressive load of 200 kN. As depicted in Figure 10b, compression was applied using a crush plate with a 75 mm radius (150 mm total diameter) at a constant speed of 1 mm/s. The same plate geometry was replicated in the analysis to enhance reliability. Separate crush plates were employed for the longitudinal (X-direction) and transverse (Y-direction) loading directions, and the analyses were performed independently for each direction.

**Figure 11:**
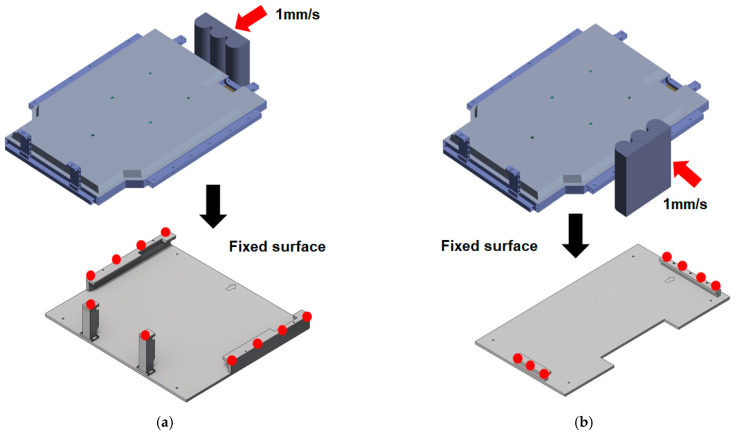
Schematic of the compression test procedure: (**a**) compression applied to the side of the BPC using cylindrical crush plates at 1 mm/s, (**b**) opposite side compression setup with identical velocity conditions.

**Figure 12:**
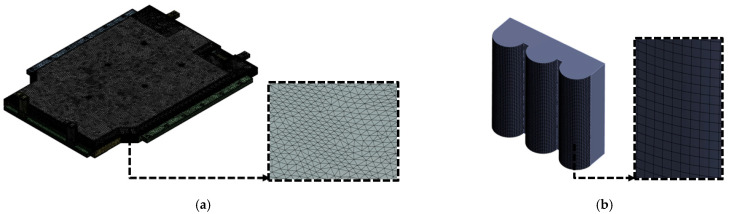
Finite element mesh models for compression simulation: (**a**) battery pack case (BPC), (**b**) cylindrical crush plate used as the compression head.

Figure 11 shows a schematic of the compression analysis procedure performed on both sides of the BPC along the X- and Y-directions. Independent loading conditions were defined for each direction, with the curved crush plate applying compressive force at a consistent rate of 1 mm/s. In the simulation, the opposite side of the BPC was constrained under fixed boundary conditions. As shown at the bottom of Figure 11, direction-specific fixed jigs were applied according to the loading direction. This configuration replicated the actual experimental constraints, thereby ensuring consistency between the simulation and the physical test setup. The crush plate was modeled as an undeformable, semi-cylindrical rigid body made of structural steel to ensure that the load was transmitted without structural deformation. The analysis scenario reproduced the actual test conditions by maintaining a constant load of 200 kN for approximately 10 min before unloading as the crush plate retracted. Figure 12 shows the finite element mesh models used for compression analysis. Figure 12a presents the complete BPC model, which utilizes a refined, high-density mesh to accurately capture the complex geometry and detailed stress transfer paths. Figure 12b depicts the curved crush plate model, designed with a radius of 75 mm and a spacing of 30 mm, enabling the simulation to account for load distribution effects induced by curvature variations during contact. Table 3 summarizes the finite element mesh information for the BPC and the crush plate used in the analysis. The BPC model consisted of 3,638,393 nodes and 1,453,197 elements, while the crush plate comprised 17,572 nodes and 5571 elements. Identical material properties, as listed in Table 2, were applied to all components to ensure material consistency throughout the model. The high-resolution mesh configuration and precise material properties enabled precise tracking of subtle contact responses and localized stress concentrations that occurred during compression. Incorporating identical experimental conditions (including compression rate, maximum load, dwell time, jig geometry, and boundary constraints) into the simulation significantly enhanced the physical validity and consistency of the analysis results. This approach enabled the researchers to validate analytical reliability through quantitative comparisons and trend analyses with experimental results, providing an essential foundation for improving predictive accuracy in designing similar structural systems for future applications.

**Figure 13:**
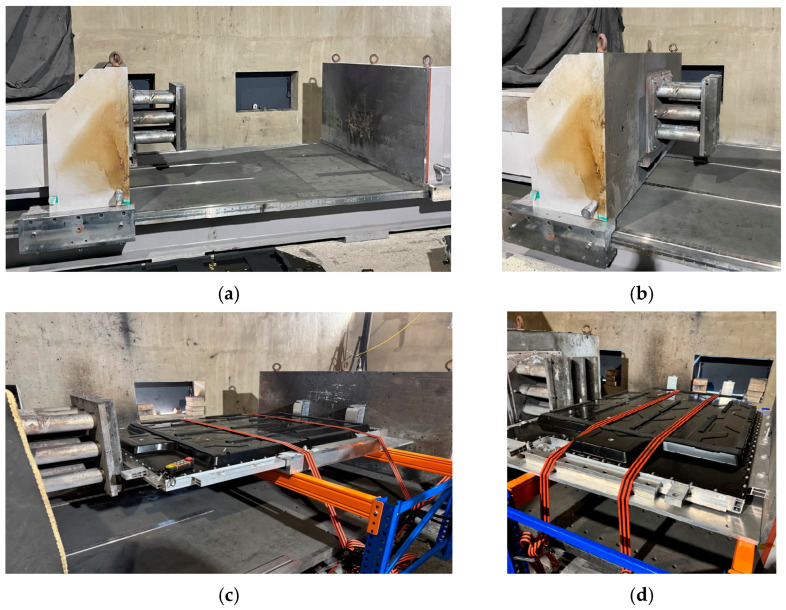
Experimental setup and test configuration for the compression test: (**a**) compression equipment installed on the testbed, (**b**) cylindrical crush plate system for applying uniform pressure, (**c**) completed BPC test setup along the X-axis direction, (**d**) completed BPC test setup along the Y-axis direction.

Figure 12 shows the finite element mesh models used for compression analysis. Figure 12a shows the complete BPC model, employing a refined, high-density mesh to accurately represent the material properties and the complex structural details. Figure 12b depicts the curved crush plate model, designed with a radius of 75 mm and a spacing of 30 mm, enabling the analysis to account for load distribution effects induced by curvature variations during contact. Table 3 summarizes the finite element mesh information of the BPC and the crush plate models used in the compression analysis. The BPC model consisted of 3,638,393 nodes and 1,453,197 elements, while the crush plate comprised 17,572 nodes and 5571 elements. This high-resolution mesh configuration provided a foundation for precisely tracking the subtle contact response and local stress concentrations that occur during compression analysis. The material properties listed in Table 2 were applied to all components to ensure consistent and reliable analysis results. In the compression analysis, the contact conditions between the crush plate and the BPC were a key variable. To realistically simulate the experimental environment, a friction coefficient of 0.2 was applied at the contact interface. This value aligns with previous experimental findings involving contact between aluminum alloy A6061-T6 and structural steel (compression plate), where a friction coefficient of approximately 0.2 has been reported in the relevant literature [51]. Applying this condition enhanced the physical validity of the analysis, resulting in a high degree of agreement with the experimental compression test results. Figure 13 includes images of the test equipment and setup used in the actual compression experiment. Figure 13a shows the hydraulic compression apparatus mounted on the test bed, consisting of a rigid load-transmission frame and a sliding actuator. Figure 13b shows the crush plate designed to apply constant surface pressure while minimizing structural eccentricity and ensuring uniform load transfer. Figure 13c,d show the BPC mounted on the fixed jig for compression testing in the X- and Y-directions, respectively, prepared under identical loading rates and boundary conditions to facilitate repeatable measurements. The experiments were conducted under the same conditions as FEA, allowing for effective cross-validation of the analysis results with experimental data. This integrated approach enabled a systematic evaluation and validation of the structural stability and stress distribution characteristics of the BPC under compression loading.

### 2.3. PSD-Based BPC Random Vibration Analysis and Test Conditions

PSD-based FEA was performed to assess the vibration durability of the EV BPC. This analysis aimed to quantitatively predict the structure’s vibration response and fatigue life by simulating the random vibration environment encountered during normal vehicle operation. Both the analysis and experiments were conducted in accordance with domestic and international vibration standards, including UL 2596 [50], IEC 62660-2 [52], and ISO 12405-3 [53]. Vibration loads were independently applied in the X-, Y-, and Z-directions. The PSD input conditions for each axis are outlined in Table 4, and the theoretical background of the vibration analysis is summarized in Equations (2)–(5) below. Equation (2) represents the system’s natural frequency *fn*, Equation (3) defines the damping ratio *ζ*, Equation (4) describes the representative frequency *fpsd* used in PSD analysis, and Equation (5) expresses the RMS acceleration (Grms) calculated by integrating the PSD. The vibration conditions were set according to frequencies and PSD levels commonly used in the automotive industry, with separate tests conducted for each axis.

(2) $$fn=\frac{1}{2\pi }\sqrt{\frac{K}{m}}$$

(3) $$\xi =\frac{C}{2\sqrt{Km}}$$

(4) $$fpsd=\frac{F^{2}}{\Delta f}$$

(5) $$Grms=\sqrt{\int_{f1}^{f2}PSD\left(f\right)df}$$

**Figure 14:**
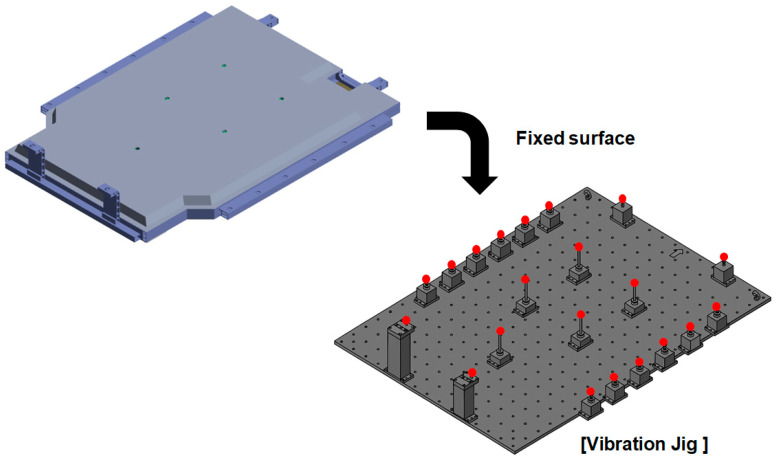
Random vibration analysis setup illustrating the BPC model mounted on a vibration jig with fixed boundary supports.

Figure 14 provides a schematic of the boundary conditions and finite element model used in the random vibration analysis. The BPC was secured on the vibration jig with fixed surface conditions applied at the same attachment points used in the actual test. These boundary conditions enhance the physical reliability of the analysis model and ensure consistency with the test setup, allowing for quantitative comparisons between simulation and test results.

**Figure 15:**
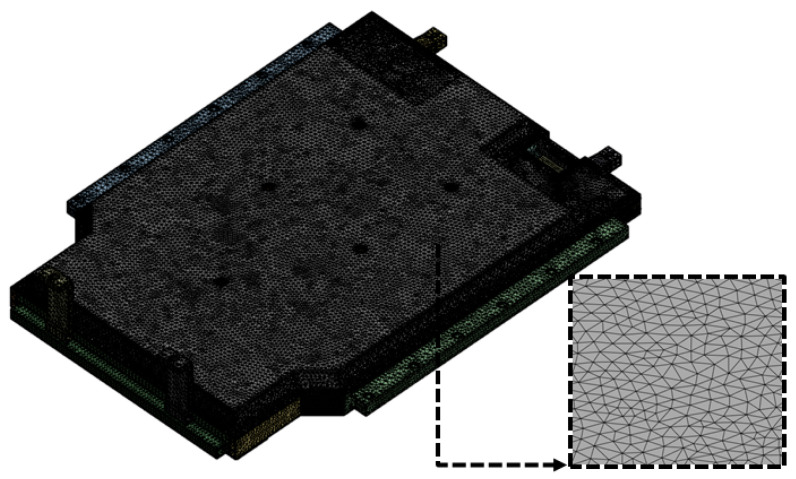
Finite element mesh model of the battery pack case (BPC) used in random vibration analysis, illustrating detailed meshing for structural evaluation.

Figure 15 shows the finite element mesh model of the BPC that was used for the random vibration analysis. A fine mesh was created based on the BPC’s geometry, accurately reflecting complex structural details and boundary shapes to precisely capture its structural response during vibration analysis. The analysis model consisted of 3,683,232 nodes and 1,672,901 elements to ensure sufficient mesh density for structural reliability and convergence. The mesh information is summarized in Table 5. Identical material properties, as listed in Table 2, were applied to all components to ensure reproducible and physically valid analysis results.

**Figure 16:**
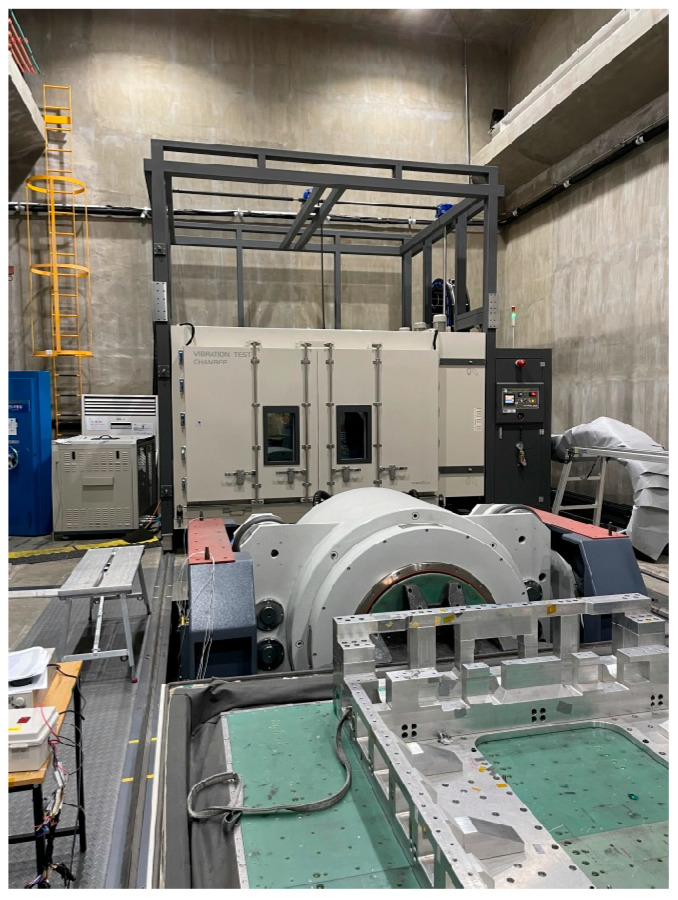
Photograph of the random vibration durability test setup, showing the vibration test equipment and slip table used for BPC specimen mounting.

**Figure 17:**
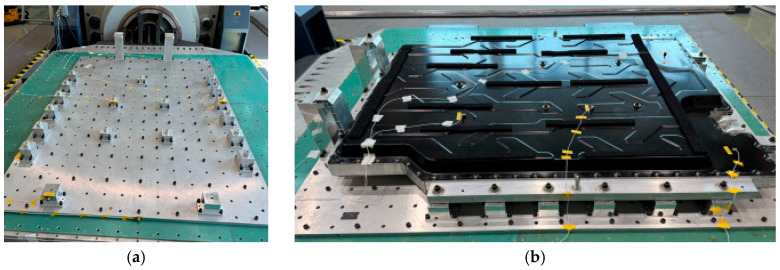
Random vibration test setup: (**a**) Vibration jig with mounting holes and fixtures; (**b**) BPC specimen mounted on the jig for testing.

The random vibration durability test was conducted to assess the structural integrity and vibration resistance characteristics of the EV BPC. As shown in Figure 16, the experiment was performed using a high-performance electrodynamic vibration testing system. The test equipment could apply a maximum shock load of 60,000 kgf, operate within a frequency range up to 1700 Hz, and achieve a maximum displacement of ±25.5 mm with an acceleration capacity of 980 m/s^2^. The detailed specifications of the vibration test system are summarized in Table 6. Figure 17b shows the BPC securely mounted on a vibration jig, which was fixed using multiple holes and fasteners, as shown in Figure 17a, to prevent any detachment or unwanted displacement during testing. Although the vibration system supports various multi-axis vibration modes, including Sine, Random, Shock, SoR, and RoR, this study employed a single-axis excitation method, applying vibration sequentially along the X-, Y-, and Z-directions. The test conditions were consistent with the boundary conditions defined in the preceding finite element analysis, enabling direct quantitative comparison between numerical and experimental results. In particular, the BPC’s vibration response characteristics were evaluated considering the jig fixation conditions, slip table properties, and correlation with the input PSD data. Vibration durability performance was validated through repeated measurements.

## 3. Structural Response Evaluation of EV BPC Through Analysis and Experimental Testing

### 3.1. Bottom Impact Analysis and Experimental Validation of BPC

**Figure 18:**
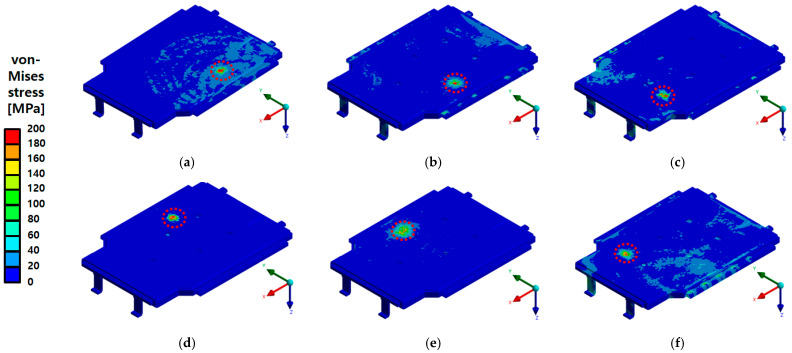
von Mises stress distribution results at six drop impact points on the full BPC assembly, including the lower cover. The red dashed line indicates the localized impact region where peak stresses occurred. Maximum stresses at each point were as follows: (**a**) Point 1—351.04 MPa, (**b**) Point 2—347.29 MPa, (**c**) Point 3—341.4 MPa, (**d**) Point 4—336.38 MPa, (**e**) Point 5—333.24 MPa, (**f**) Point 6—316.99 MPa. Although these peak stresses locally exceeded the yield strength of the A6061-T6 aluminum alloy (245 MPa), the resulting plastic deformation was confined to small dented regions around the impact areas. No cracking, coolant leakage, or exposure of the battery modules was observed under any of the drop conditions.

**Figure 19:**
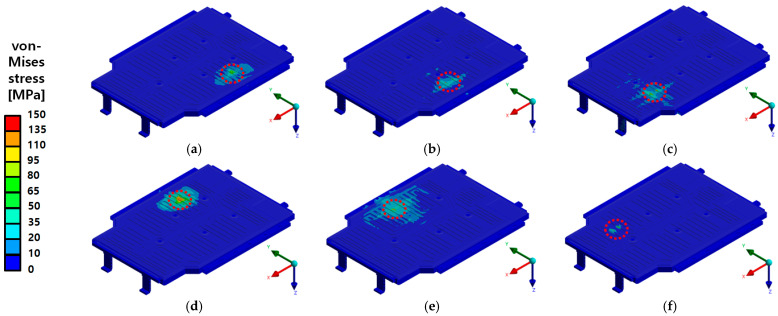
von Mises stress distribution results at six drop impact points for the intermediate BPC assembly excluding the lower cover and incorporating the internal cooling channel (A3003-O). The red dashed line indicates the localized impact region. Maximum stress values were measured as follows: (**a**) Point 1—138.13 MPa, (**b**) Point 2—121.08 MPa, (**c**) Point 3—116.52 MPa, (**d**) Point 4—124.65 MPa, (**e**) Point 5—110.13 MPa, (**f**) Point 6—106.06 MPa. Compared to the full assembly, the stress distribution was more uniform and reduced, confirming the effectiveness of internal components in stress dispersion.

Figure 18 and Figure 19 present the analysis results for the bottom impact conditions of the BPC when subjected to an upper impact load. The results illustrate the von Mises stress distribution for the full assembly model and the intermediate assembly model, excluding the lower cover, respectively. The full assembly model (Figure 18) shows local stress concentrations at the impact locations, with maximum stress reaching approximately 351.04 MPa at Point 1, which exceeds the nominal yield strength of the A6061-T6 aluminum alloy (245 MPa). In the corresponding drop tests, shallow dents and local plastic deformation were observed around the impact areas, but no cracking of the BPC, loosening of joints, or coolant leakage occurred. Points 1–3, impacted by a cylindrical impactor, exhibited relatively higher stress levels, whereas Points 4–6, impacted by a spherical impactor, showed a wider but less intense stress distribution, resulting in slightly lower maximum stress values. In the intermediate assembly model excluding the lower cover (Figure 19), the inclusion of internal structures, such as cooling channels, led to a more uniform stress distribution, with the maximum stress decreasing to approximately 138 MPa. This reduction indicates that the impact load was effectively distributed through various internal components. These findings demonstrate that the BPC’s stress distribution characteristics are significantly influenced by the impact location, impactor geometry, and the combination of internal components, and that the bottom structure primarily behaves as an energy-absorbing layer that allows limited plastic denting while maintaining overall structural integrity.

**Figure 20:**
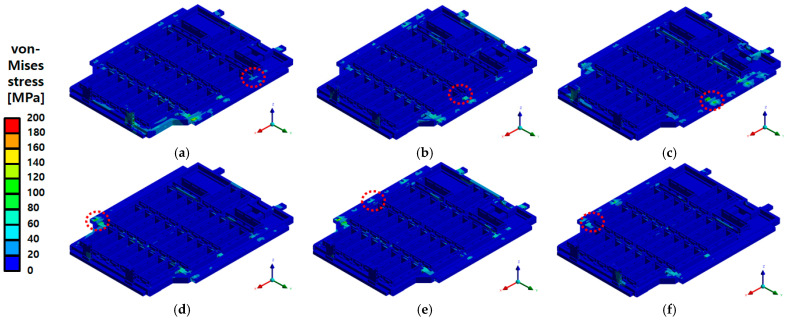
von Mises stress distribution results at six drop impact points for the BPC frame component model observed from the underside. The red dashed line indicates the localized impact region. Each result reflects the stress response under a lower impact (drop) test condition: (**a**) Point 1: Max 150.51 MPa; (**b**) Point 2: Max 122.56 MPa; (**c**) Point 3: Max 113.22 MPa; (**d**) Point 4: Max 109.43 MPa; (**e**) Point 5: Max 102.67 MPa; (**f**) Point 6: Max 94.82 MPa. All stress values remained below the yield strength of A6N01S-T6 aluminum alloy (240 MPa), indicating that structural failure did not occur under the tested drop conditions.

**Figure 21:**
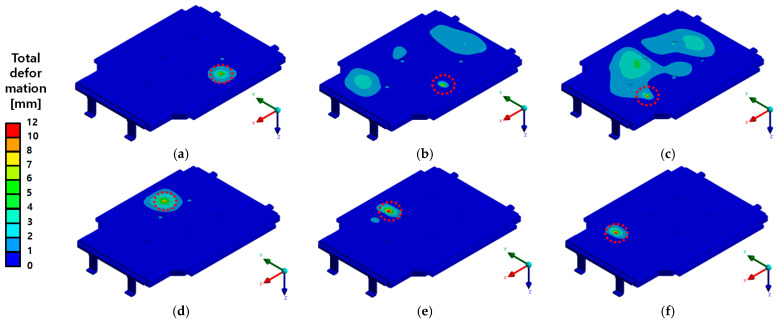
Total deformation results (in mm) at six drop impact points for the full BPC model including the lower cover. The red dashed line indicates the localized impact region. Maximum deformation was observed at (**a**) Point 1: Max 11.73 mm; (**b**) Point 2: Max 10.45 mm; (**c**) Point 3: Max 10.05 mm; (**d**) Point 4: Max 8.75 mm; (**e**) Point 5: Max 8.34 mm; and (**f**) Point 6: Max 6.94 mm. Displacement was mostly concentrated at the center and rear areas of the upper panel, particularly under cylindrical impact conditions.

Figure 20 and Figure 21 present the results of the bottom impact analysis performed at six impact locations on the BPC, showing the von Mises stress distribution and total deformation distribution, respectively. Figure 20 shows the von Mises stress distribution in the BPC’s structural frame and internal components after impact, allowing a visual assessment of the stress concentration areas at each impact point. Relatively high stress concentrations were observed in the side frame and rib structures, which can be attributed to differences in local impact load transfer paths and structural sensitivity depending on the impact location. Figure 21 shows the maximum total deformation results for the full BPC model (including the lower cover), corresponding to the same setup as Figure 18. Across all impact locations, deformation tended to concentrate around the center or rear plane of the upper panel. For Points 1–3, the maximum deformation occurred at Point 1, reaching approximately 11.73 mm. In contrast, for Points 4–6, the maximum deformation of approximately 6.94 mm was observed at Point 4, which was attributed to differences in impact energy dispersion characteristics depending on the impactor geometry and impact location. The obtained stress and deformation responses exhibit strong sensitivity to impact location, geometric configuration, and material distribution, providing foundational data for identifying structural weaknesses and optimizing design reinforcements in future BPC designs.

**Figure 22:**
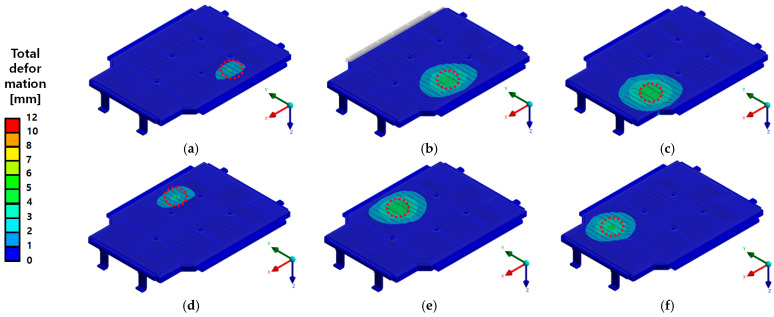
Total deformation results (in mm) at six drop impact points for the BPC configuration excluding the bottom cover and including the internal cooling channel. The red dashed line indicates the localized impact region. The maximum deformation was (**a**) Point 1: Max 5.99 mm; (**b**) Point 2: Max 4.82 mm; (**c**) Point 3: Max 4.23 mm; (**d**) Point 4: Max 5.02 mm; (**e**) Point 5: Max 4.17 mm; and (**f**) Point 6: Max 3.35 mm. This configuration demonstrated a more even stress distribution and reduced deformation compared to the full assembly.

Figure 22 shows the total deformation distribution obtained from six drop impact points for the BPC assembly model, excluding the lower cover, thereby exposing the internal cooling channel. The analysis results indicate that, for all bottom impact points, the maximum deformation was predominantly concentrated around the center of the upper panel, with the largest deformation measured at Point 1 (approximately 5.99 mm). In contrast, Point 6 exhibited a relatively lower deformation of about 3.35 mm, demonstrating variations in structural response according to impact location and geometric differences. Although overall stiffness was partially reduced due to the absence of the lower cover, the results suggest that the internal cooling channel’s reinforcement effect and the redistribution of load transfer paths contributed to a more uniform stress distribution. This analysis of deformation behavior provides essential data for evaluating the design contributions of individual components and for establishing lightweight design and reinforcement strategies for structural optimization.

**Figure 23:**
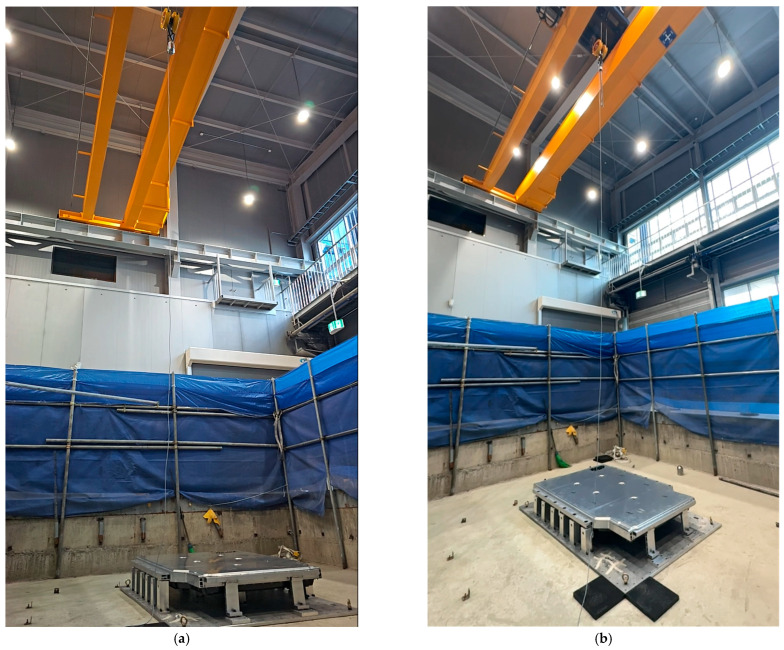
Test setup for the bottom impact test of the lower cover of the BPC using two different impactor types: (**a**) Setup for the spherical bottom impact test; (**b**) setup for the cylindrical bottom impact test.

Figure 23 shows the test setup for the bottom impact test of the BPC lower cover using two different impactor types (spherical and cylindrical). Figure 23a shows the setup with a spherical impactor, while Figure 23b shows the setup with a cylindrical impactor, describing the experimental setup designed to compare and analyze differences in the structural response according to impactor geometry. The test equipment includes a crane system and impact guides installed to ensure accuracy and energy reproducibility at each impact location, thereby enhancing the overall reliability of the experimental results.

**Figure 24:**
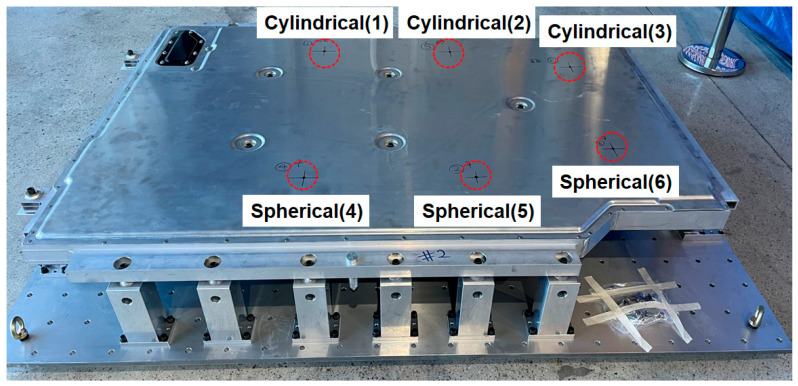
Designated impact locations on the lower cover of the BPC: cylindrical bottom impact weights applied at Points 1–3 and spherical bottom impact weights at Points 4–6.

**Figure 25:**
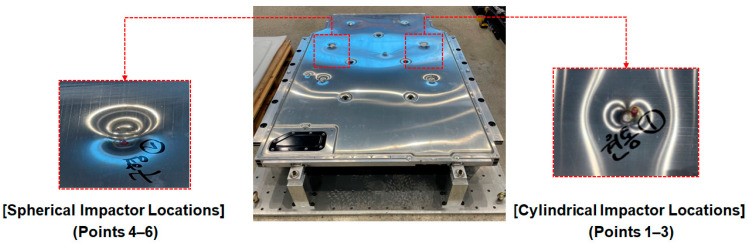
Photographic evidence of impact marks left on the BPC lower cover after drop tests: (**Left**) spherical impactor marks at Points 4–6; (**Right**) cylindrical impactor marks at Points 1–3. The handwritten Korean annotations visible on the specimen indicate dent measurement points recorded during the physical testing.

Figure 24 and Figure 25 show the impact locations and the resulting physical impact marks observed on the lower cover of the BPC during bottom impact testing. Figure 24 shows six impact locations, with Points 1–3 subjected to a cylindrical impactor and Points 4–6 subjected to a spherical impactor. These configurations serve as baseline data for analyzing the differences in structural response according to impactor geometry. Figure 25 shows the impact marks formed on the lower cover after testing. Broad and shallow deformation patterns were observed at Points 4–6 (impacted by the spherical impactor), whereas narrower and deeper localized deformations occurred at Points 1–3 (impacted by the cylindrical impactor). These differences were attributed to variations in impact load concentration and correspond closely with the stress concentration and deformation distribution predicted in the numerical analysis. Additionally, no fracture or penetration damage occurred at any impact location, indicating that the lower cover maintains a structurally safe level of durability. These results experimentally demonstrate that impact location and impactor geometry directly influence the damage patterns and structural vulnerability, providing crucial reference data for establishing component reinforcement and geometric optimization strategies in future structural designs.

**Figure 26:**
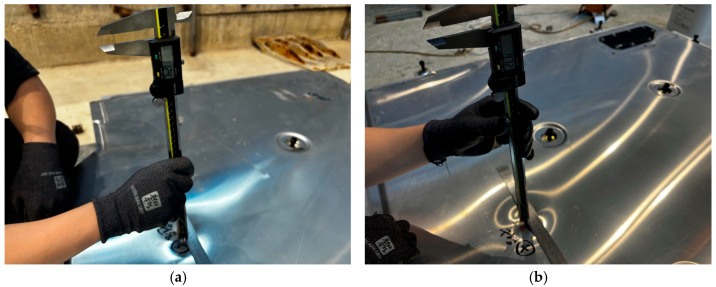
Measurement of residual dent depth at impact locations on the BPC lower cover after bottom impact tests: (**a**) Measurement after cylindrical bottom impact; (**b**) Measurement after spherical bottom impact. Handwritten Korean annotations visible on the specimen indicate dent measurement points recorded during the physical testing.

Figure 26 shows images of the residual dent depth measurements taken at each impact location on the BPC lower cover after bottom impact testing using a digital caliper. Figure 26a shows the measurement process for dents caused by the cylindrical impactor, while Figure 26b shows the process for measuring dents induced by the spherical impactor. The results indicate that dents produced by the cylindrical impactor were relatively deeper than those produced by the spherical impactor. This observation aligns with the stress concentration and localized deformation patterns previously identified in the numerical analysis and visual observation of impact marks. These quantitative measurements of actual deformation support the reliability of the simulation results and provide a clearer understanding of how structural response varies depending on the geometry of the impactor. In conclusion, measuring residual deformation serves as an effective method for validating consistency between numerical analyses and experimental results and provides foundational data for establishing impact-resistant design criteria and damage limit evaluations for future BPC development.

**Figure 27:**
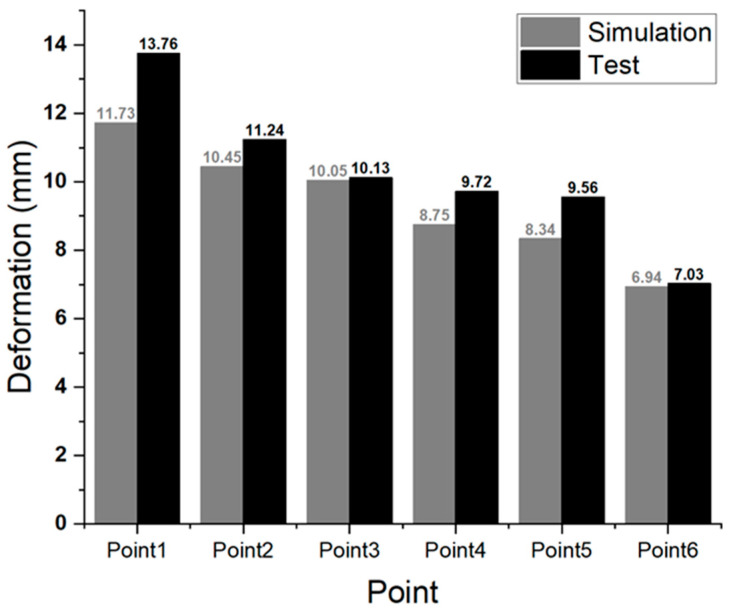
Comparison of residual dent depth between simulation and lower impact test results at six impact points on the BPC lower cover.

Table 7 summarizes the residual dent depth measurements following the bottom impact test on the BPC lower cover, and Figure 27 quantitatively compares the deformation obtained from numerical analysis with the experimental results. The test conditions were based on the major bottom impact test standards specified in MOLIT, GB/T 31467.3, and UN 38.3 (10 kg-impactor with a drop height of 2 m–5 m). However, to simulate a more severe impact scenario, the drop height was increased to 7 m. The test results showed residual indentation depths of ≤13.76 mm at all impact points, satisfying the structural safety criteria and earning a “Pass” rating for all test locations. Furthermore, as shown in Figure 27, the deformation responses from the numerical simulation and experimental test exhibited generally similar trends, with an average deviation within ±1.0 mm, thereby validating the reliability of the simulation model. These results experimentally validate the effectiveness of numerical simulation-based predictions for structural response characteristics under bottom impact test conditions and suggest that simulation-based design validation can effectively contribute to ensuring actual structural reliability.

**Figure 28:**
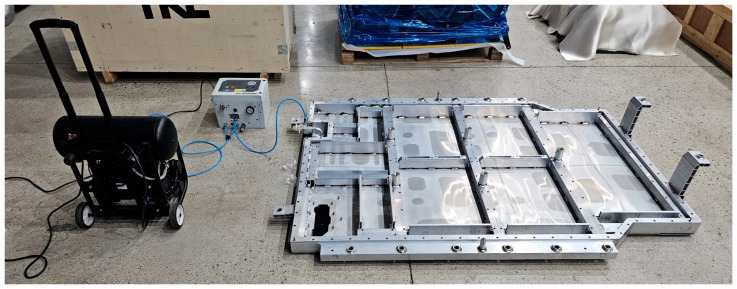
Post-impact air leakage test setup for verifying sealing integrity of the BPC cooling channel after bottom impact.

**Figure 29:**
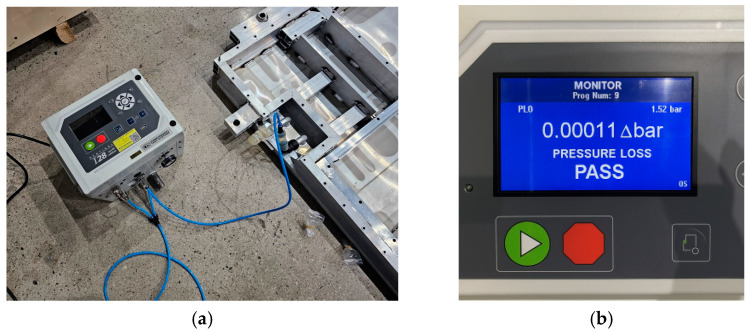
Close-up views of the air leakage test after bottom impact: (**a**) Setup of connection and pressurization equipment; (**b**) display showing the test result with measured pressure loss.

Figure 28 and Figure 29 show the verification test procedure used to evaluate the sealing integrity of the BPC cooling channel following the bottom impact test. This test was conducted to assess the potential for damage to the cooling channel caused by structural impacts from the impactor (which could affect cooling performance) and to comprehensively validate the structural reliability of the system. Figure 28 shows the equipment setup for the leakage test. The test was carried out with the fluid passages inside the structure configured identically to those of the actual cooling system, enabling evaluation of possible leakage throughout the entire BPC cooling channel. Pressure supply and sealing devices were connected to the inlet and outlet ports. Internal pressure was applied, and leakage was determined by monitoring whether the pressure was maintained for a specified duration. Figure 29a provides a close-up view of the test setup, and Figure 29b shows an image of the screen displaying the pressure retention state during the actual test. The test was performed in accordance with standard leakage assessment procedures. After applying the designated internal pressure to the cooling channel, the pressure retention was monitored for 20 s. A slight pressure loss of approximately 0.00011 bar was observed. However, this variation was considered within the margin of error, indicating no functional leakage in the cooling channel. These results provide key evidence that the cooling channel did not sustain structural damage following the bottom impact test and that its sealing integrity and cooling performance can be maintained under real-world conditions.

### 3.2. Compression Analysis and Experimental Validation of BPC

**Figure 30:**
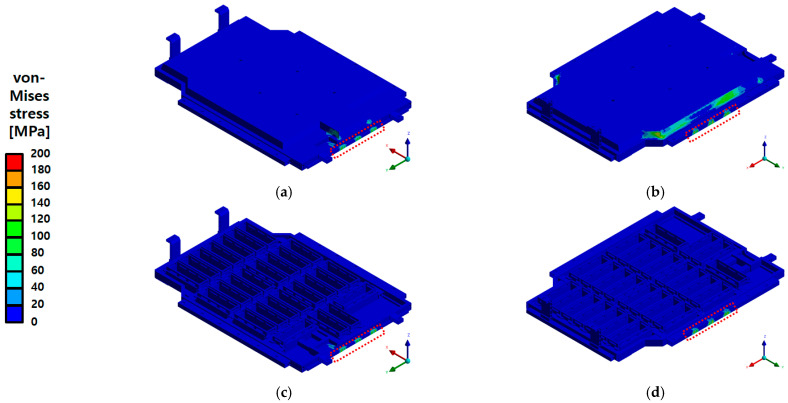
von Mises stress distribution of the battery pack case (BPC) under side compression: (**a**) X-axis compression with upper cover—Max 294.47 MPa; (**b**) Y-axis compression with upper cover—Max 362.39 MPa; (**c**) X-axis compression without upper cover—Max 294.47 MPa; (**d**) Y-axis compression without upper cover—Max 362.39 MPa. The red dashed line indicates the region where the maximum stress occurs under the applied side compression.

In this study, FEA was conducted to evaluate the structural stiffness and stability of the EV BPC under lateral compression loads. The accuracy and reliability of the numerical results were then verified through actual compression tests. The analysis was performed under four conditions, considering the presence of the upper cover and the loading direction (X-direction and Y-direction) as variables. According to the analysis results presented in Figure 30, the maximum von Mises stress was 294.47 MPa during X-direction compression and 362.39 MPa during Y-direction compression with the upper cover installed. These values are lower than the yield strengths of the main structural aluminum alloys, A6N01S-T6 and A6061P (240 MPa and 245 MPa, respectively; see Table 2), indicating a sufficient safety margin without structural failure. Although some reinforcement frames were made of SGACUD 60/60, which has a relatively lower yield strength of 142.66 MPa, the areas where localized stress concentrations occurred overlapped with the structurally reinforced areas and did not result in failure. Even without the upper cover (Figure 30c,d), the maximum stress levels remained similar at 294.47 MPa in the X-direction and 362.39 MPa in the Y-direction. The stress distribution exhibited localized concentrations along the rib structures within the internal frame. However, these stresses did not exceed the yield strength and therefore did not compromise the overall structural integrity. These findings suggest that the upper cover has a limited impact on the overall compressive stiffness, with the primary structural resistance largely reliant on the internal frame and lower base structure.

**Figure 31:**
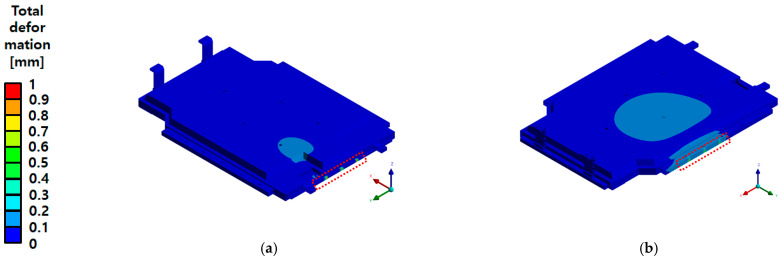
Total deformation distribution of the battery pack case (BPC) under side compression: (**a**) X-axis compression with upper cover—Max 0.81 mm; (**b**) Y-axis compression with upper cover—Max 0.98 mm. The red dashed line indicates the location of maximum deformation generated during the side compression load.

**Figure 32:**
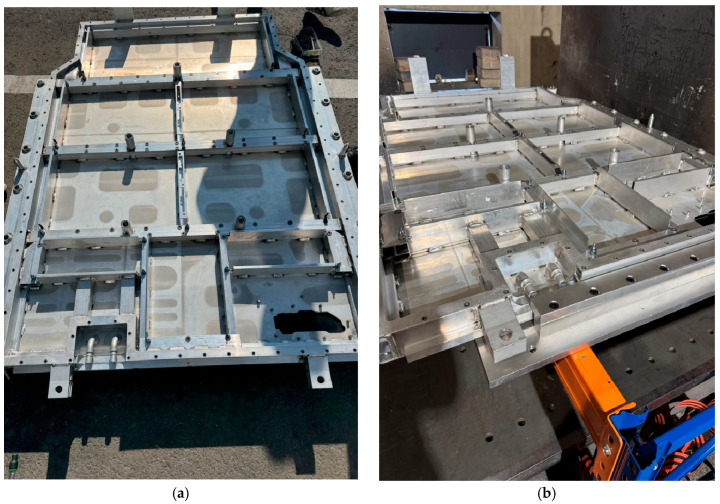
Post-test internal condition of the battery pack case (BPC) after compression: (**a**) View in the X-axis direction; (**b**) View in the Y-axis direction. No visible deformation or structural damage was observed.

As shown in the total deformation results in Figure 31, the maximum deformation under X-direction compression with the upper cover installed was 0.81 mm, while that under Y-direction compression was 0.98 mm. This sub-millimetre deformation, relative to the overall external dimensions of the BPC, demonstrates that the structural integrity was successfully maintained. The compression test was conducted under the same boundary conditions as the FEA. A curved compression plate was moved at a rate of 1 mm/s to apply the load, which was then held for 10 min after the reaction force reached 200 kN before being released. After the test, the BPC was disassembled to inspect the internal structure (Figure 32). No fractures or significant deformations were observed in the frame structure, including the ribs and support members.

**Figure 33:**
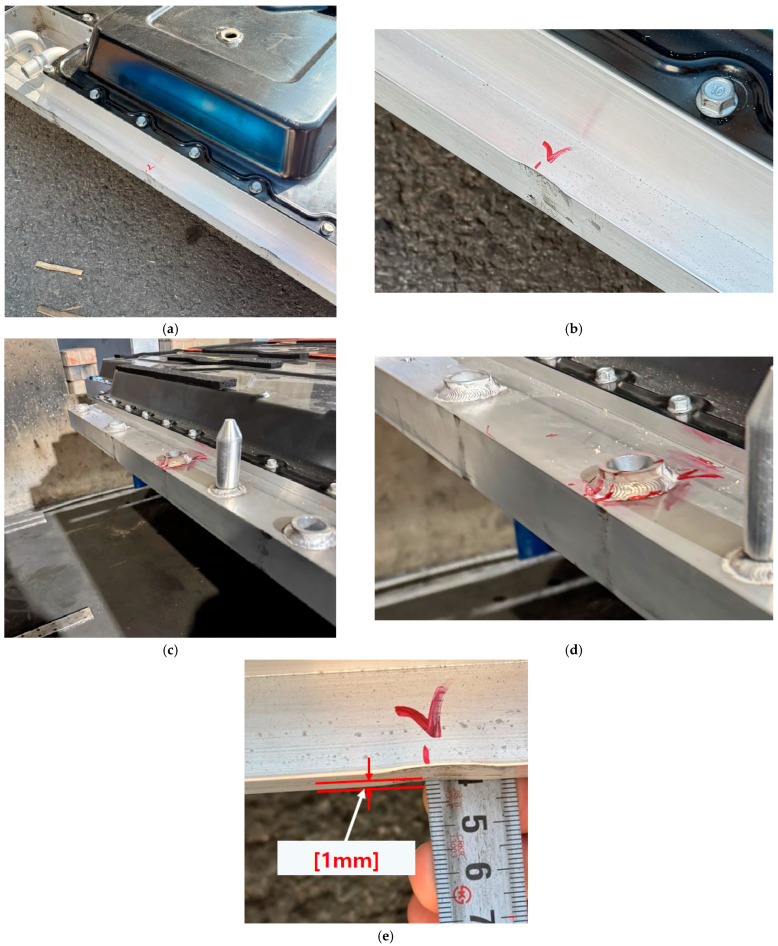
Visual inspection of deformation on the battery pack case (BPC) after compression tests: (**a**) Enlarged view of the X-axis side showing visual surface marks; (**b**) Enlarged view of the Y-axis side showing visual surface marks; (**c**) Close-up image of the X-axis side indicating minor deformation; (**d**) Close-up image of the Y-axis side indicating minor deformation; (**e**) Direct measurement of the maximum lateral permanent deformation (~1 mm) using a steel ruler at the most deflected region.

Figure 32 and Figure 33 show the results of inspecting the exterior and internal structures after the compression tests. Figure 32 presents the internal frame immediately after testing with the upper cover removed. No cracks, weld separations, or noticeable bending deformations were found in the ribs or support members, indicating that the BPC maintained structural stability during side compression. Figure 33 shows magnified views of local surface indentations and marks on the outer frame after the X- and Y-direction compression tests, respectively. In particular, the direct measurement at the most deflected region in Figure 33e indicates a permanent lateral indentation of approximately 1.0 mm. These shallow, localized deformations are consistent with the numerical predictions in Figure 31. For the Y-direction compression case, the finite element analysis predicts a maximum deformation of 0.98 mm, which differs from the experimentally measured 1.0 mm by only about 2%. This small discrepancy confirms good agreement between the simulation and the test, and the remaining deformation level is negligible compared with the overall dimensions of the BPC. These compression responses can be explained by the load-path characteristics of the BPC structure. Under lateral compression, the closed-section side frame and multiple cross-members distribute the applied load along the longitudinal and transverse beams. As a result, most of the compressive energy is carried by combined membrane and bending actions in the outer plates, while the inner ribs and support members remain predominantly within the elastic range. This structural behavior causes only localized yielding at the direct contact region between the compression plate and the outer skin, appearing as minor surface indentations in Figure 33, whereas global buckling or progressive collapse of the frame does not occur. Therefore, the observed deformation pattern represents a desirable failure-safe response in which local outer-panel deformation absorbs the compressive load while preserving the overall integrity of the BPC. Additionally, although the numerical analysis showed that the difference in overall structural stiffness between the cases with and without the upper cover was small, the upper cover helped redistribute the compressive load more uniformly over the surface opposite to the applied force, thereby mitigating local stress concentrations and acting as a supplementary protective layer. Collectively, the analytical and experimental results confirm that the proposed BPC structure satisfies the “no rupture’’ requirement specified in UL 2596 and GB/T 31467.3, demonstrating sufficient structural durability and stability even under abnormal lateral compressive loads. These findings also suggest that the structural design can serve as a safety-oriented platform for diverse operating environments, including multidirectional impact and thermal-runaway conditions.

### 3.3. Vibration Analysis and Experimental Validation of BPC

**Figure 34:**
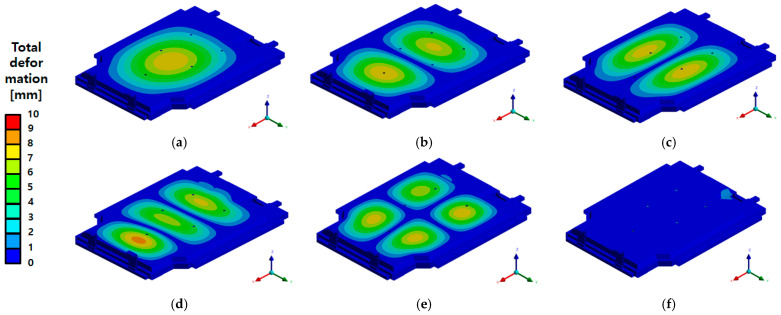
Total deformation contours of the battery pack case (BPC) under random vibration analysis, presented for six modal frequencies: (**a**) Mode 1 at 22.094 Hz; (**b**) Mode 2 at 42 Hz; (**c**) Mode 3 at 51.951 Hz; (**d**) Mode 4 at 71.34 Hz; (**e**) Mode 5 at 74.718 Hz; (**f**) Mode 6 at 94.607 Hz. The maximum deformation was observed in Mode 4 with 8.43 mm displacement.

In this study, finite element vibration analysis was conducted based on the PSD conditions presented in Table 4, and the structural integrity of the BPC was comprehensively evaluated through corresponding experimental validation. The vibration analysis was performed as a linear static analysis step to determine the BPC’s natural frequencies and quantitatively assess its structural response characteristics and vibration sensitivity. Figure 34 shows the total deformation distributions for the natural frequencies derived from the six vibration modes, visually representing the BPC’s periodic response behavior. Resonance vibration responses were observed at 22.094 Hz for Mode 1, 42 Hz for Mode 2, and 51.951 Hz for Mode 3. Notably, Mode 4 exhibited a maximum deformation of 8.43 mm at 71.34 Hz, indicating that vertical (Z-direction) resonance is a primary factor affecting the entire structure, with the deformation concentrated at the center of the upper cover. Subsequently, in Mode 5 (74.718 Hz) and Mode 6 (94.607 Hz), the vibration responses became more distributed, showing localized resonance characteristics in other regions of the structure. Among these, Mode 4 represents the frequency with the maximum response and serves as a key mode for evaluating structural safety. It can also be used as a reference value to define input criteria for future impact and vibration durability analyses (random vibration analyses). The following section compares the analytical results obtained under actual PSD conditions with the experimental results to examine the stability and durability of the BPC structure.

**Figure 35:**
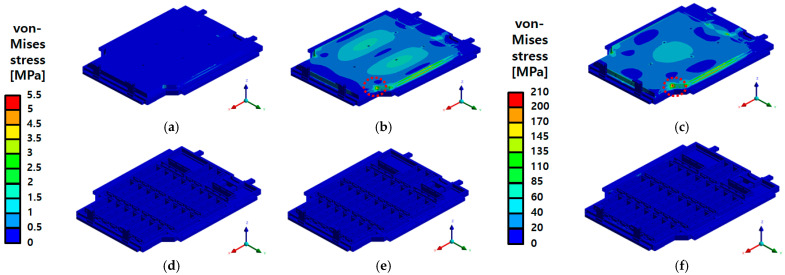
von Mises stress distribution of the battery pack case (BPC) under random vibration analysis: (**a**) X-axis—Max 1.51 MPa; (**b**) Y-axis—Max 5.44 MPa; (**c**) Z-axis—Max 208.59 MPa; (**d**) X-axis (Inner)—Max 1.51 MPa; (**e**) Y-axis (Inner)—Max 5.44 MPa; (**f**) Z-axis (Inner)—Max 208.59 MPa. The red dashed line indicates the region where the maximum stress response is concentrated under random vibration loading.

**Figure 36:**
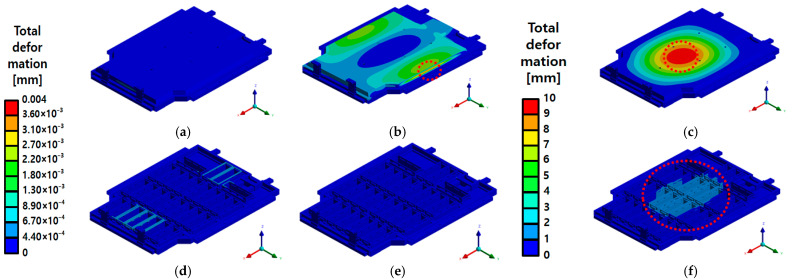
Total deformation distribution of the battery pack case (BPC) under random vibration analysis: (**a**) X-axis—Max 0.0011 mm; (**b**) Y-axis—Max 0.0035 mm; (**c**) Z-axis—Max 9.39 mm; (**d**) X-axis (Inner)—Max 0.0011 mm; (**e**) Y-axis (Inner)—Max 0.0035 mm; (**f**) Z-axis (Inner)—Max 9.39 mm, with the red dashed line indicating the region of maximum deformation.

A comprehensive evaluation of the BPC’s structural integrity was performed based on the response analysis and experimental results obtained under actual PSD conditions. The results from the random vibration analysis indicated minimal stress distributions along the X- and Y-directions, suggesting a negligible structural impact. However, higher stress levels were observed along the Z-direction. Notably, the maximum von Mises stress along the Z-direction reached 208.59 MPa, which remains below the yield strength of the aluminum alloy (A6061-T6) used, indicating a low likelihood of structural damage. As shown in Figure 35, the overall stress distribution across the structure appeared relatively uniform, with limited local stress concentrations. A similar trend was noted in the deformation results. While the maximum deformations along the X- and Y-directions were extremely small, measured at 0.0011 mm and 0.0035 mm, respectively, local deformation along the Z-direction reached up to 9.39 mm. This behavior is attributed to the concentration of vibration energy at the center of the upper plate in the Z-direction, leading to resonant behavior. The results in Figure 36 confirm that such deformations do not affect the structure’s overall functionality, demonstrating that the vibration load was effectively distributed throughout the structure. Therefore, the random vibration analysis indicates that the EV BPC can maintain structural stability under multi-axial vibration environments while also suggesting the need for additional reinforcement and design optimization to mitigate resonance behavior in the Z-direction.

**Figure 37:**
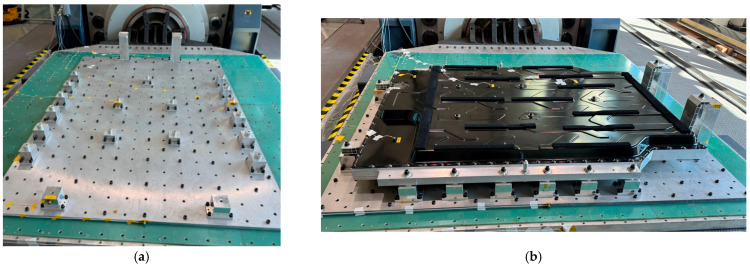

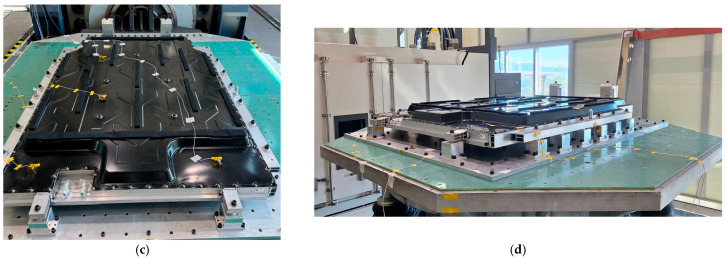
Experimental setup and visual observation of the battery pack case (BPC) for random vibration and compression tests: (**a**) Customized jig for random vibration testing; (**b**) Setup completed for X-axis compression test; (**c**) Setup completed for Y-axis compression test; (**d**) Test completed for Z-axis compression.

To validate the consistency of the numerical analysis results, an experimental vibration test was conducted using the actual BPC. As shown in Figure 37, custom jigs were fabricated for random vibration testing along the X-, Y-, and Z-directions. These jigs were precisely installed on the test equipment to ensure accurate transmission of vibration responses in all directions. The BPC was then mounted vertically for each loading direction, and all bolts were fully tightened before initiating the tests. The same setup procedure was applied sequentially for every axial direction.

**Figure 38:**
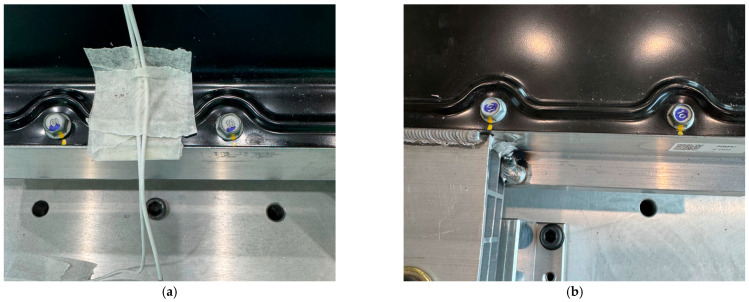
Visual inspection of external bolt loosening on the battery pack case (BPC) before and after random vibration test: (**a**) Initial marking applied for displacement and rotation tracking; (**b**) Bolted joint state after testing without observable loosening or rotation.

In addition, Figure 38 provides a visual inspection of the external bolted joints before and after the random vibration test, confirming that no loosening or rotation occurred. This result verifies the fastening stability of the BPC and supports the reliability of the experimental vibration procedure.

The experimental results obtained using visual markings were found to be consistent with the vibration analysis results presented in Figure 34, Figure 35 and Figure 36. While the analysis indicated a maximum overall deformation of 8.43 mm and relatively high stress levels along the Z-direction, no physical changes, such as detachment or rotation, were observed at the external fastening points after testing in all three directions (X, Y, and Z). This demonstrates that the BPC maintained sufficient stiffness and fastening stability within the design parameters, thereby empirically confirming its structural reliability against vibration loads under real-world operating conditions. Furthermore, the consistency between the simulation-based design validation and durability assessment results with the experimental findings supports the validity of simulation-based prediction methods for ensuring structural integrity in future design processes.

**Figure 39:**
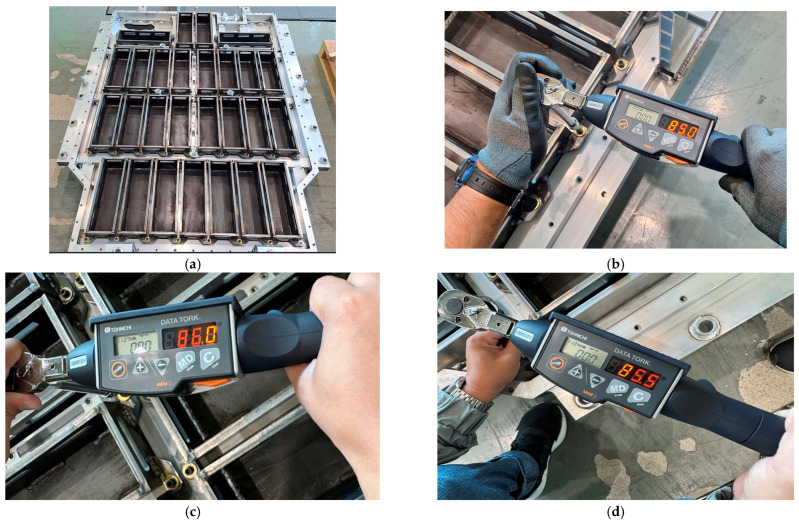
Post-test inspection and torque measurement of the battery pack case (BPC) after random vibration and compression testing: (**a**) Internal structure of BPC after all tests; (**b**) Measured torque of bolts after X-axis compression test; (**c**) Measured torque after Y-axis compression test; (**d**) Measured torque after Z-axis compression test.

As shown in Figure 39, a post-test inspection was conducted using a digital torque gauge to quantitatively assess bolt loosening in the internal structure of the EV BPC after the random vibration tests were completed. The reference tightening torque was set at 90 Nm, and the measured average values after testing ranged from approximately 84.5 Nm to 86.0 Nm. This reduction rate, within about 6%, falls well within the generally acceptable ±10% tolerance range for mechanically fastened structures, indicating that no structurally significant bolt loosening occurred. The internal structure of the BPC is crucial since it is where the battery cells are mounted, requiring higher structural reliability than the exterior. Thus, these quantitative inspections are highly important. Post-test torque checks were performed for the Z-, Y-, and X-direction tests, all showing a high retention rate of approximately 93.9% relative to the reference torque, confirming stable fastening conditions across all directions. Comparing these measurement results with earlier analyses (Figure 34, Figure 35 and Figure 36) shows that stresses and displacements concentrated on structural members did not significantly impact the internal fasteners during testing. This provides strong evidence supporting the reliability and accuracy of the simulation-based design. Moreover, since the test was conducted under high-vibration conditions that exceeded the ISO 12405-3 and IEC 62660-2 vibration test standards, the results demonstrate that the BPC structure can maintain sufficient reliability and safety in real operating environments. Thus, the test results confirm consistency between simulation-based design validation and measurement-based evaluation, providing crucial evidence for the validity and reproducibility of simulation-based predictions aimed at securing structural integrity in the future.

## 4. Conclusions

This study evaluated the mechanical durability and structural integrity of an EV BPC by combining FEA with physical structural testing. The primary analysis focused on bottom impact, lateral compression loads, and PSD-based random vibration analysis, which facilitated a quantitative assessment of the BPC’s structural responses under various operating conditions. The bottom impact analysis identified primary stress concentrations at the corners of the lower frame, and the deformation patterns closely aligned with experimental results, thereby validating the reliability of the simulation model. The compression analysis revealed that the maximum stresses in both the X- and Y-directions remained within the yield strengths of the respective materials, ensuring sufficient structural safety margins. While the presence or absence of the upper cover had minimal impact on overall stress distribution, it served a supplementary role in mitigating local stress concentrations and acted as a protective component. In the random vibration tests, the highest response was observed in the Z-direction, with strong agreement between simulation predictions and experimental outcomes. Post-test qualitative inspections using visual markings confirmed that there was no loosening or rotational displacement of any bolts, indicating that fastening integrity was maintained even under complex vibrational conditions. Overall, these analyses demonstrate that the proposed BPC structure offers adequate stiffness and durability to withstand various mechanical loads encountered in real-world scenarios, including underbody impacts, lateral compression, and road-induced vibrations. Notably, the bottom impact analysis confirmed the structure’s capability to absorb shock and dissipate energy without damaging key components. The compression analysis recorded total deformations below 1 mm, highlighting the design’s effectiveness as a high-strength, lightweight structure. Additionally, the vibration analysis confirmed the effectiveness of the resonance prevention design, as no displacements or loosening of fastened joints were noted, ensuring fastening reliability during high-speed driving and prolonged operational conditions. The reliability of the FEM-based structural analysis technique utilized in this study was validated by its alignment with physical test results, indicating its potential for enhancing cost and time savings as a simulation-based design validation methodology. Furthermore, the integrated structural reliability assessment process, which combines simulation and testing, has the potential to serve as a foundational technology for thorough safety assessments in complex environments, such as those involving thermal runaway, collision-induced fires, and moisture ingress. Future work will focus on extending the experimental methodology to include multi-directional and high-speed impact tests, fatigue and durability evaluations under temperature-dependent conditions, and full-vehicle-level vibration verification to further refine the correlation between FEA and experimental data. In addition, analyses considering thermal–mechanical interactions will be conducted to evaluate how temperature variations during thermal runaway events influence the structural response of the BPC. The effects of bolt preload relaxation, gasket behavior, and joint stiffness on the overall mechanical response will also be experimentally investigated to improve the predictive accuracy of fastening integrity models. Moreover, advanced material studies—such as hybrid aluminum–composite configurations and flame-retardant surface treatments—will be pursued to optimize the balance between lightweight design and safety performance. These continued efforts are expected to contribute to the development of next-generation EV battery pack cases with enhanced mechanical robustness, thermal safety, and long-term reliability.

## Figures and Tables

**Table 1:** Mechanical properties of materials used in the EV battery pack case (BPC) components.

Material	Density [kg/m^3^]	Young’s Modulus [GPa]	Yield Strength [MPa]	Poisson’s Ratio
SGACUD 60/60	7900	210	142.66	0.3
PA6-GF30	1360	9.7	30	0.35
A3003-O	2730	68	61	0.3
A6082S-T6	2700	68	260	0.3
A6N01S-T6	2700	68	240	0.3
A6061P	2700	68	245	0.3
Structure Steel	7850	200	250	0.3

**Table 2:** Mesh information of the finite element models used in the bottom impact simulation.

Model	Node	Elements
BPC model	852,867	1,436,879
Cylindrical weight	27,600	25,074
Spherical weight	15,865	86,443

**Table 3:** Mesh information of the finite element models used in the compression test simulation, including the BPC model and crush plate.

Model	Node	Elements
BPC Model	3,638,393	1,453,197
Crush Plate	17,572	5571

**Table 4:** Power spectral density (PSD) data applied in vibration analysis and testing for X, Y, and Z axes [54].

X Axis		Y Axis		Z Axis	
Frequency (Hz)	PSD (G^2^/Hz)	Frequency (Hz)	PSD (G^2^/Hz)	Frequency (Hz)	PSD (G^2^/Hz)
5	0.0125	5	0.04	5	0.05
10	0.03	10	0.04	10	0.06
20	0.03	20	0.04	20	0.06
200	0.00025	200	0.0008	200	0.0008
Grms	0.96 g	Grms	1.23 g	Grms	1.44 g

**Table 5:** Mesh information of the finite element model used in the random vibration simulation for the BPC.

Model	Node	Elements
BPC Model	3,683,232	1,6727,901

**Table 6:** Specifications of the vibration test system used for random and shock durability tests, including excitation force, frequency range, and control modes.

Category	Specification
Max Force	Sine: 30,000 kgf
	Random: 24,000 kgf
	Shock: 60,000 kgf
Frequency Range	5–1700 Hz
Displacement	±25.5 mm
Acceleration	Up to 980 m/s^2^
Payload Capacity	Up to 6000 kg
Slip Table	Size: 2500 × 2500 mm
	Hole Type: M8
	Hole Pitch: 100 mm
Control System	16-channel controller
	Modes: Sine, Random, Shock, SoR, RoR

**Table 7:** Residual dent depth measurements and qualification results after bottom impact tests at six designated impact points on the BPC lower cover.

Classification	Point 1	Point 2	Point 3	Point 4	Point 5	Point 6
Depth (mm)	13.76	11.24	10.13	9.72	9.56	7.03
Result	Pass	Pass	Pass	Pass	Pass	Pass

## Data Availability

The original contributions presented in this study are included in the article. Further inquiries can be directed to the corresponding authors.
